# DNA methylation testing with S5 for triage of high‐risk HPV positive women

**DOI:** 10.1002/ijc.34050

**Published:** 2022-05-24

**Authors:** Rachael Adcock, Belinda Nedjai, Attila T. Lorincz, Dorota Scibior‐Bentkowska, Rawinder Banwait, Norah Torrez‐Martinez, Michael Robertson, Jack Cuzick, Cosette M. Wheeler, New Mexico HPV Pap Registry Steering Committee

**Affiliations:** ^1^ Centre for Cancer Prevention, Wolfson Institute of Population Health Queen Mary University of London London UK; ^2^ Center for HPV Prevention University of New Mexico Comprehensive Cancer Center Albuquerque New Mexico USA

**Keywords:** cervical cancer, DNA methylation, high‐risk HPV, molecular markers, triage

## Abstract

Methylation of host and viral genes is promising for triage of women with high‐risk human papillomavirus infections (hrHPV). Using a population‐based sample of hrHPV positive women with cervical biopsies within 12 months after cervical screening, the clinical value of the S5 methylation classifier (S5), HPV genotyping and cytology were compared as potential triage tests, for outcomes of cervical intraepithelial neoplasia (CIN) grade 3 or greater (CIN3+), CIN2+ and CIN2, and the area under the curve (AUC) calculated. S5 scores increased with histopathology severity (*P*_trend_ < .001). For CIN3+, the AUC was 0.780 suggesting S5 provides good discrimination between <CIN3 and CIN3+. AUCs were significant for all pairwise comparisons of <CIN2, CIN2 and CIN3+ (*P* < .001). The positive predictive value (PPV) of HPV16/18 genotyping for women with any abnormal cytology was greater than S5 (25.36% vs 20.87%, *P* = .005) for CIN3+, while sensitivity was substantially greater for S5 (83.33% vs 59.28%, *P* < .001). Restricting to women with abnormal cytology, but excluding those with high‐grade cytology, both S5 and HPV16/18 provided CIN3+ PPVs high enough to recommend colposcopy. Triage with S5 also appeared useful for hrHPV positive women negative for HPV16/18 (CIN3+ PPV: 7.33%, sensitivity: 57.52%). S5 provided increased sensitivity for CIN3+ compared to HPV16/18 genotyping for hrHPV positive women, overall and when restricted to women with abnormal cytology, suggesting S5 may improve colposcopy referral. S5 also has the ability to distinguish between <CIN2, CIN2 and CIN3+, a finding of importance for managing CIN2, given the complexity and uncertainty associated with this diagnosis.

## Graphical abstract

What's new?

Human papillomavirus (HPV) testing is a more sensitive but less specific approach for cervical cancer screening than cytology. Methylation testing of host and viral genes has emerged as a promising approach for the triage of women with high‐risk HPV. In this population‐based study, using the DNA methylation S5 classifier as a triage test for high‐risk HPV‐positive women yields significantly greater sensitivity and similar positive predictive values for CIN3+ detection than HPV genotyping or cytology triage. Furthermore, S5 can improve discrimination between low‐ and high‐grade cervical precancer, providing valuable information in the often complex and uncertain diagnosis of CIN2.

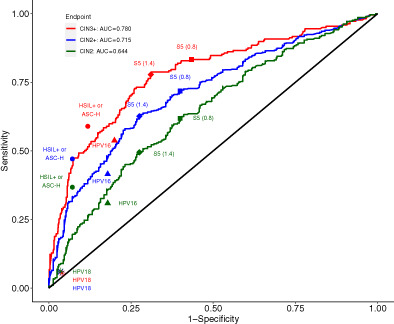

**Abbreviations**
ASC‐H: atypical squamous cells cannot exclude HSIL
ASC‐US: atypical squamous cells of undetermined significance
AUC: area under the curve
CIN: cervical intraepithelial neoplasia
HPV: human papillomavirus
hrHPV: high‐risk human papillomavirus
HSIL: high‐grade squamous intraepithelial lesion
LBC: liquid based cytology
LSIL: low‐grade squamous intraepithelial lesion
NMHPVPR: New Mexico HPV Pap Registry
NPV: negative predictive value
PPV: positive predictive value

## 1 INTRODUCTION

It is well established that human papillomavirus (HPV) testing is a more sensitive screening test than cytology. It allows earlier diagnosis of high‐grade disease, and is more effective at preventing invasive cervical cancers.^1^, ^2^, ^3^ However, HPV testing is less specific than cytology and most HPV positive women have transient infections which will regress naturally.^4^ Thus many colposcopy referrals and associated cervical excisional treatments are unnecessary and could be reduced with better triage tests. Conversely, triage also produces decisions about which women with HPV infection and/or abnormal cytology do not need referral to an expert clinician, which can result in loss to follow‐up and undetected cancers.

Various triage strategies have been suggested, but currently no optimal approach has been identified. Previously, cytology was the primary screening method in many countries, with cytology results of high‐grade squamous intraepithelial lesions or greater (HSIL+) or atypical squamous cells, cannot exclude HSIL (ASC‐H) recommended for referral to immediate colposcopy.^5^ However, for women with less severe cytological abnormalities, including low‐grade squamous intraepithelial lesions (LSIL) or high‐risk HPV (hrHPV) positive atypical squamous cells of unknown significance (ASC‐US), the best management approach remains uncertain. Referral of all women with LSIL or hrHPV positive ASC‐US cytology is not efficient; the proportion of women found to have cervical intraepithelial neoplasia (CIN) grade 2 or greater (CIN2+) at colposcopy has varied significantly, with generally only a small proportion of colposcopies showing detectable high‐grade disease.^6^, ^7^, ^8^

Triage tests for women with hrHPV infections that have greater accuracy, reliability and reproducibility remain an important challenge. Cytology, HPV genotyping, HPV viral load, immunocytochemistry using p16^ink4a^ alone or p16/ki‐67 dual‐staining, as well as DNA methylation, have been suggested as potential triage tests for hrHPV positive women.^9^, ^10^, ^11^, ^12^, ^13^, ^14^ High levels of DNA methylation have been shown to be associated with persistent HPV infection, more severe precancerous lesions and an increased risk of cervical cancer.^15^, ^16^, ^17^, ^18^, ^19^, ^20^ Measuring DNA methylation of host and viral genes at specific CpG sites has emerged as a promising approach for distinguishing between potentially progressive CIN2/3 lesions and those likely to regress.^18^, ^21^, ^22^, ^23^, ^24^ In a recent meta‐analysis including 43 studies of 16 336 women, DNA methylation of multiple human and viral genes, especially the HPV L1 and L2 gene regions of HPV16, were found to be significantly increased in women with both CIN2+ and CIN3+ biopsies compared to those with ≤CIN1, and provided an increased sensitivity and similar specificity, compared to ASC‐US or worse cytology (ASC‐US+).^25^

Molecular triage tests are advantageous as they are less subjective and can be performed on multiple specimen types, including vaginal self‐samples^26^ and urine,^27^ as morphologically intact cells are not required. However, to‐date no single gene, human or viral, has shown high enough sensitivity to be the sole triage marker. Identifying an optimum panel of markers remains a key area of interest. We have previously developed a DNA methylation classifier (S5) based on target regions of the human gene *EPB41L3*, and HPV late gene regions (L1, L2) of HPV16, HPV18, HPV31 and HPV33.^19^, ^20^, ^21^, ^28^ S5 has shown promise for the accurate detection of CIN2+, CIN3+ and cancers in many studies worldwide including Canada, China, Colombia, Finland, Mexico and the United Kingdom.^15^, ^16^, ^20^, ^29^, ^30^, ^31^

Here we conducted the first population‐based study in the United States evaluating the clinical value of the S5 DNA methylation classifier, compared to liquid based cytology (LBC) or HPV genotyping, for triage of hrHPV positive women.

## 2 MATERIALS AND METHODS

### 2.1 Study population

Women attending routine cervical screening by cytology or a combination of cytology and HPV testing in New Mexico, with and without cervical biopsies taken within 12 months of a screening cytology were identified at three major laboratories serving New Mexico residents between June 2014 and December 2015 (n = 128 649). Women were aged 17 to 82 years. Although no statewide organised screening program exists, coverage has been shown to be reasonably high.^32^ Inclusion criteria included women whose screening cytology immediately preceding their biopsy was hrHPV positive for one or more of 13 hrHPV genotypes (HPV16, 18, 31, 33, 35, 39, 45, 51, 52, 56, 58, 59 and 68; N = 4112). Cytology and histology classifications were obtained from the New Mexico HPV Pap Registry (NMHPVPR) and were based on community laboratory results and pathologist diagnoses. Technicians performing methylation were blinded to cytology and histology results.

A stratified sample of LBC specimens was selected to over‐represent women who developed high‐grade lesions (CIN2+). In total 798 LBC specimens from hrHPV positive women were selected for S5 DNA methylation. A further 159 LBC specimens from women positive for one of seven hrHPV types (HPV16, 18, 31, 33, 45, 52 and 58) and cytology negative who were not biopsied within 12 months were also tested using S5 to further examine specificity.

Figure S1 shows the consort diagram and distribution of histologic findings for the population and the sample. To account for the selection bias towards high‐grade lesions in the sample, sampling weights were applied so that the results better represent the entire biopsied screening population.

### 2.2 HPV genotyping

hrHPV positivity was determined using the Linear Array HPV Genotyping assay (Roche Diagnostics, USA) on LBC samples. The Linear Array assay individually detects 37 HPV types including the 13 high‐risk types. Full details have been described previously.^33^

### 2.3 DNA methylation classifier

DNA was extracted from aliquots of the LBC samples with the QIAamp DNA mini kit (QIAGEN, Germany) following the manufacturer's instructions. Bisulfite conversion of 200 ng of genomic DNA was performed using the EZ DNA methylation kit (Zymo Research, USA). We used previously optimised polymerase chain reaction (PCR) conditions for the markers included in the S5 classifier. The S5 classifier uses the mean of the percent of DNA methylated at each CpG site in the promoter region of *EPB41L3* (CpG sites 425, 427 and 438 relative to transcription start site) and viral regions of HPV16 (L1: CpG sites 6367, 6389), HPV18 (L2: CpG sites 4257, 4262, 4266, 4269, 4275, 4282), HPV31 (L1: CpG sites 6352, 6354) and HPV33 (L2: CpG sites 5557, 5560, 5566, 5572), as well as the proportion of CpG sites methylated for HPV16 L2 (CpG sites 4238, 4247, 4259, 4268, 4275).^28^ Amplification was performed using the PyroMark PCR kit (QIAGEN, Germany) with 20 ng input of converted DNA in a 25 μL volume following manufacturer's instructions. The PCR products were pyrosequenced using a PyroMarkQ96 ID (QIAGEN, Germany) instrument. All pyrosequencing runs included negative and positive controls of known methylation levels (0%, 50% and 100%) to allow standardised direct comparisons between different primer sets and all runs were replicated twice.

### 2.4 Statistical analysis

The predefined S5 DNA methylation classifier was calculated as

$$S5=\left(30.9*\mathrm{EPB}41L3\right)+\left(13.7*\mathrm{HPV}16_{L1}\right)+\left(4.3*\mathrm{HPV}16_{L2}\right)+\left(8.4*\mathrm{HPV}18_{L2}\right)+\left(22.4*\mathrm{HPV}31_{L1}\right)+\left(20.3*\mathrm{HPV}33_{L2}\right)$$

A cut‐point of ≥0.8 was used for methylation positivity in the major analyses, as has been previously validated, and full details on the marker have been published.^20^, ^28^ An additional exploratory S5 cut‐point of ≥1.4 was used as identified by Youden's Index as a potential optimum cut‐off based on maximising the sum of the sensitivity and specificity for CIN3+ detection.^34^ Descriptive statistics for age, cytology, genotype positivity and S5 methylation were calculated. HPV genotypes were ranked hierarchically when multiple types were present; all HPV16 positive, HPV18 positive if HPV16 negative, HPV31/33 positive if HPV16 and 18 negative, positive for any other hrHPV type if negative for HPV16, 18, 31 and 33. Only the highest ranking HPV member of the hierarchy was included in the representations. Trends in median S5 scores were assessed by age and biopsy grade. Histograms were plotted showing the distribution of S5 scores and genotype by histology (<CIN2, CIN2, CIN3/adenocarcinoma in situ [AIS] and cancer).

The diagnostic accuracy of the S5 classifier for histologic outcomes was calculated for hrHPV positive biopsied women with abnormal cytology. Positive predictive value (PPV), negative predictive value (NPV), sensitivity and specificity were estimated for outcomes of CIN3+, CIN2+ and CIN2 for cytology, HPV genotyping and S5. We do however note calculating specificity for CIN3+ assumes that CIN2 is a false positive. Receiver operator characteristic (ROC) curves for S5 were plotted for CIN3+, CIN2+ and CIN2 and area under the curve (AUC) calculated.

Finally, for hrHPV positive women with abnormal cytology excluding HSIL+ and ASC‐H, the diagnostic accuracy for combinations of HPV16, 18, 31 and 33 and S5 methylation were considered, with a focus on the value of using S5 or HPV16/18 as a second triage test for women initially negative for the other.

Analyses were re‐weighted to represent all women attending routine cervical screening in New Mexico in the study time period who were biopsied within 12 months based on histologic diagnoses (Figure S1). All estimates, confidence intervals and *P*‐values were based on adjusted analyses to account for re‐weighting and McNemar *P*‐values were used to compare accuracy of triage tests. All analyses were conducted in Stata 16.1 and R Studio version 1.3.1073.

## 3 RESULTS

LBC from a total of 798 women attending routine cervical screening who were hrHPV positive and biopsied within 12 months was assessed using the S5 DNA methylation classifier, and weighted to represent 4112 hrHPV positive women in the population (Figure S1); weighted numbers are shown in subsequent descriptions unless otherwise noted. The median age of women was 33 years (interquartile range [IQR] 27‐42), with 66.34% aged ≥30 years (Table 1).

**TABLE 1:** Descriptive statistics for hrHPV positive women attending routine cervical screening in New Mexico, and biopsied within 12 months of enrolment cytology, re‐weighted to represent hrHPV positive biopsied women (n = 4112)

	All (N = 798; n^a^ = 4112)	Histology						
		Negative (N = 121; n^a^ = 1866)	CIN1 (N = 159; n^a^ = 1238)	CIN2 (N = 298; n^a^ = 541)	CIN3 (N = 201; n^a^ = 426)	AIS (N = 11; n^a^ = 22)	Cancer (N = 8; n^a^ = 19)	CIN3+ (N = 220; n^a^ = 467)
*Age (years)*								
Median (IQR)	33 (27‐42)	36 (29‐46)	32 (27‐41)	31 (25‐39)	31 (28‐37)	35 (29‐40)	41 (31‐63)	*32 (28‐39)*
<30 years n (%)^b^	1384 (33.66)	509 (27.28)	459 (37.08)	240 (44.36)	167 (39.20)	6 (27.27)	2 (10.53)	*175 (37.47)*
≥30 years n (%)^b^	2728 (66.34)	1357 (72.72)	779 (62.92)	301 (55.64)	259 (60.80)	16 (72.73)	17 (89.47)	*292 (62.53)*
*Cytology n (%)* ^b^								
Negative	315 (7.66)	216 (11.58)	78 (6.30)	13 (2.40)	6 (1.41)	2 (9.09)	0 (0.00)	*8 (1.71)*
ASC‐US	1797 (43.70)	971 (52.04)	514 (41.52)	185 (34.20)	119 (27.93)	8 (36.36)	0 (0.00)	*127 (27.19)*
LSIL	1217 (29.60)	478 (25.62)	553 (44.67)	142 (26.25)	42 (9.86)	0 (0.00)	2 (10.53)	*44 (9.42)*
ASC‐H	379 (9.22)	123 (6.59)	47 (3.80)	96 (17.74)	104 (24.41)	2 (9.09)	7 (36.84)	*113 (24.20)*
AGC	117 (2.85)	78 (4.18)	15 (1.21)	7 (1.29)	6 (1.41)	8 (36.36)	3 (15.79)	*17 (3.64)*
HSIL+	287 (6.98)	0 (0.00)	31 (2.50)	98 (18.11)	149 (34.98)	2 (9.09)	7 (36.84)	*158 (33.83)*
*Genotyping n (%)* ^b^								
HPV16 positive	1004 (24.42)	370 (19.83)	210 (16.96)	169 (31.24)	227 (53.29)	14 (63.64)	14 (73.68)	*255 (54.60)*
HPV18 positive	222 (5.40)	77 (4.13)	85 (6.87)	35 (6.47)	21 (4.93)	4 (18.18)	0 (0.00)	*25 (5.35)*
HPV31/33 positive	494 (12.01)	169 (9.06)	156 (12.60)	96 (17.74)	68 (15.96)	2 (9.09)	3 (15.79)	*114 (15.63)*
other hrHPV positive	2392 (58.17)	1250 (66.99)	787 (12.60)	241 (44.55)	110 (25.82)	2 (9.09)	2 (10.53)	*114 (24.41)*
*S5 methylation*								
Median (IQR)	0.75 (0.48‐2.87)	0.68 (0.43‐1.46)	0.67 (0.46‐1.81)	1.39 (0.58‐5.78)	5.97 (1.47‐10.57)	2.22 (0.94‐11.98)	22.59 (16.07‐26.60)	*6.37 (1.48‐10.99)*
≥0.8 cut‐off n (%)^b^	1961 (47.68)	756 (40.07)	483 (38.99)	336 (62.10)	352 (82.63)	18 (82.24)	16 (86.57)	*387 (82.77)*
≥1.4 cut‐off n (%)^b^	1489 (36.21)	493 (26.16)	366 (29.56)	271 (50.01)	326 (76.64)	16 (72.89)	16 (86.57)	*359 (76.87)*

Abbreviations: AGC, atypical glandular cells; AIS, adenocarcinoma in situ; ASC‐H, atypical squamous cells—cannot rule out HSIL; ASC‐US, atypical squamous cells of undetermined significance; CIN1, cervical intraepithelial neoplasia grade 1; CIN2, cervical intraepithelial neoplasia grade 2; CIN3, cervical intraepithelial neoplasia grade 3 [CIN3] or greater [CIN3+]; hrHPV, high‐risk human papillomavirus; HSIL, high‐grade squamous intraepithelial lesion or greater [HSIL+]; IQR, interquartile range; LSIL, low‐grade squamous intraepithelial lesion.

^a^ Estimated numbers weighted to represent hrHPV positive women biopsied in the population.

^b^ Column percent.

Table 1 shows the cross‐tabulations of cytology, HPV genotype and methylation positivity by histology. Ninety‐two percent (n = 3797) of biopsied hrHPV positive women had abnormal cytology; 1797 (43.70%) women had ASC‐US cytology, 1217 (29.60%) had LSIL, 379 (9.22%) had ASC‐H, 117 (2.85%) had atypical glandular cells (AGC) and 287 (6.98%) had HSIL+ cytology. In total, 1008 (24.51%) women were diagnosed with CIN2+, of which 541 (53.67%) were CIN2 and 467 (46.33%) were CIN3+. Nineteen women were diagnosed with cancer (8 before re‐weighting [0.46%]) making estimates for cancer unreliable.

### 3.1 S5 DNA methylation and disease severity

**FIGURE 1:**
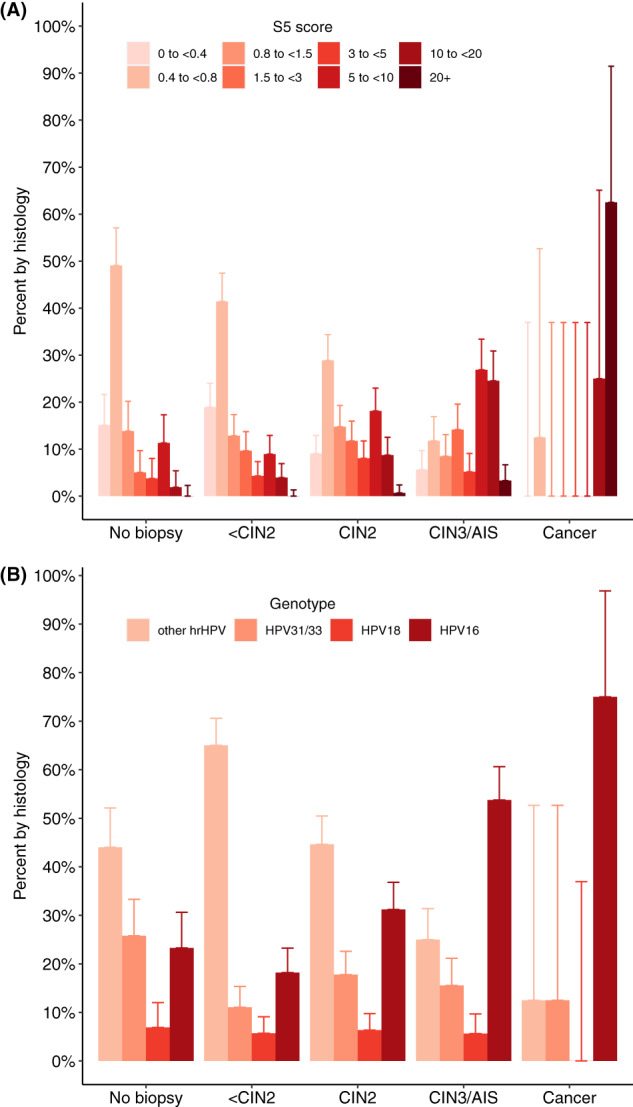
Histogram of (A) S5 score and (B) genotype by histological outcome (no biopsy within 12 months, <CIN2, CIN2, CIN3/AIS and cancer) for hrHPV positive women with screening cytology. Percentages add to 100% in each histology group. A histogram showing (A) the distribution of S5 scores (categorised) by histology (no biopsy within 12 months, <CIN2, CIN2, CIN3/AIS and cancer) and (B) distribution of genotypes (grouped) by histology. Upper 95% confidence interval (CI) are plotted. Percentage of S5 score and genotype sum to 100% within each histology group. Higher histology categories are associated with greater proportions of higher S5 score and more higher‐risk genotypes. AIS, adenocarcinoma in situ; CIN2, cervical intraepithelial neoplasia grade 2; CIN3, cervical intraepithelial neoplasia grade 3; hrHPV, high‐risk human papillomavirus

In hrHPV positive women, the median S5 score was 0.75 (IQR 0.48‐2.87), and 47.68% were positive at the ≥0.8 cut‐off (Table 1). S5 methylation showed a highly significant increasing trend with histopathology severity (*P*_trend_ < .001). Median S5 scores were 0.68 for women with a negative biopsy and 0.67 for those with CIN1, but increased to 1.39 for CIN2, 5.97 for CIN3 and 22.59 for cancer. Median S5 scores were significantly increased for each pairwise histology group comparison between <CIN2, CIN2, CIN3/AIS and cancer (*P* < .001 for each comparison). For CIN3 women, 82.63% were S5 positive, and 86.57% (seven out of eight women, unweighted) of women with cancer were S5 positive (Table 1). For the one woman with cancer not identified by S5, the S5 score was 0.71 and positivity was observed for HPV39, an HPV type for which the S5 classifier has not been optimised. Figure 1 shows the distribution of S5 scores (Figure 1A) and HPV genotyping (Figure 1B) by histology grade.

Abnormal cytology was detected in 3797 women. Of the 459 CIN3+ cases in women with abnormal cytology, 382 were S5 positive at the ≥0.8 cut‐off, corresponding to a sensitivity of 83.33% and specificity of 60.06% for <CIN2 (Table 2). For CIN2+ the S5 sensitivity was 71.84%. Notably, the sensitivity of S5 to detect CIN2 was lower than CIN3+ (61.86% vs 83.33%, *P* < .001). At a 1.4 cut‐off, which was the optimum cut‐off for a CIN3+ vs <CIN3 comparison in these data based on Youden's index, the sensitivity of S5 for CIN3+ was 77.82% and the PPV was 25.63% (Table 2). Sensitivity for CIN2+ was 62.65% with specificity for <CIN2 of 72.44%.

**TABLE 2:** Univariate diagnostic accuracy for triage using cytology, HPV genotyping or S5 methylation for CIN3+, CIN2+ and CIN2 endpoints, among hrHPV positive women with abnormal cytology re‐weighted to represent all hrHPV positive biopsied women (n = 3797)

Triage marker	Cytology					Genotyping					Methylation	
	HSIL+	AGC	ASC‐H	LSIL	ASC‐US	HPV16 positive	HPV18 positive	HPV16/18 positive	HPV31/33 positive	hrHPV other positive	S5 positive (≥0.8 cut‐off)	S5 positive (≥1.4 cut‐off)
n positive	287	117	379	1217	1797	913	159	1072	484	2241	1831	1393
CIN3+ (n = 459)												
n	157	17	113	45	127	247	25	272	72	115	382	357
PPV (95% CI)	54.94 (49.18, 60.70)	14.34 (7.98, 20.70)	29.79(25.18, 34.39)	3.68(2.62, 4.73)	7.05(5.87, 8.23)	27.01(24.13, 29.89)	15.87(10.18, 21.55)	25.36(22.76, 27.97)	14.91 (11.74, 18.08)	5.11(4.20, 6.02)	20.87(19.01, 22.73)	25.63(23.33, 27.92)
NPV (95% CI)	91.42 (90.49, 92.35)	87.99(86.94, 89.04)	89.89(88.88, 90.90)	83.96(82.54, 85.37)	83.40(81.77, 85.03)	92.65(91.70, 93.60)	88.09(87.03, 89.14)	93.15(92.20, 94.09)	88.34(87.24, 89.43)	77.89(75.83, 79.95)	96.11(95.26, 96.97)	95.77(94.96, 96.57)
Sensitivity(95% CI)	34.34(28.84, 39.83)	3.65(0.25, 7.05)	24.63(20.30, 28.97)	9.76(8.09, 11.43)	27.62(25.55, 29.69)	53.78(50.55, 57.02)	5.49(1.95, 9.04)	59.28(56.33, 62.22)	15.74(12.50, 18.99)	24.98(23.19, 26.78)	83.33(81.63, 85.04)	77.82(75.63, 80.00)
Specificity(95% CI)	96.13(93.90, 98.36)	97.01(93.91, 100.00)	92.02(89.30, 94.75)	64.88(62.20, 67.56)	49.96(47.65, 52.27)	80.04(77.44, 82.63)	96.00(92.95, 99.05)	76.04(73.48, 78.59)	87.66(84.73, 90.59)	36.30(34.31, 38.30)	56.59(54.32, 58.86)	68.97(66.54, 71.40)
CIN2+ (n = 987)												
n	256	24	209	186	312	410	58	468	167	352	709	618
PPV (95% CI)	89.14(85.53, 92.74)	20.57(13.23, 27.90)	55.16(50.15, 60.16)	15.31(13.29, 17.33)	17.35(15.60, 19.10)	44.91(41.68, 48.13)	36.45(28.96, 43.93)	43.65(40.68, 46.62)	34.41(30.18, 38.64)	15.72(14.22, 17.23)	38.71(36.48, 40.94)	44.40(41.79, 47.01)
NPV (95% CI)	79.16(77.82, 80.51)	73.84(72.42, 75.26)	77.25(75.84, 78.65)	68.97(67.18, 70.75)	66.24(64.17, 68.32)	80.00(78.54, 81.46)	74.46(73.05, 75.88)	80.96(79.48, 82.43)	75.24(73.77, 76.71)	59.22(56.78, 61.66)	85.86(84.32, 87.40)	84.67(83.23, 86.11)
Sensitivity(95% CI)	25.89(20.82, 30.96)	2.43(0.00, 5.23)	21.20(17.08, 25.31)	18.88(16.68, 21.08)	31.60(29.45, 33.75)	41.55(38.35, 44.75)	5.86(2.21, 9.52)	47.41(44.42, 50.40)	16.88(13.54, 20.22)	35.71(33.72, 37.69)	71.84(69.78, 73.90)	62.65(60.11, 65.19)
Specificity(95% CI)	98.89(97.68, 100.00)	96.70(93.46, 99.94)	93.95(91.55, 96.35)	63.32(60.61, 66.02)	47.14(44.83, 49.45)	82.10(79.61, 84.58)	96.41(93.51, 99.30)	78.51(76.05, 80.97)	88.70(85.88, 91.52)	32.79(30.85, 34.74)	60.06(57.82, 62.31)	72.44(70.10, 74.79)
CIN2^a^ (n = 528)												
n	98	7	96	142	185	163	33	196	94	238	327	261
PPV (95% CI)	75.89(68.51, 83.27)	7.27(2.18, 12.36)	36.13(30.36, 41.90)	12.08(10.21, 13.94)	11.08(9.58, 12.59)	24.52(21.25, 27.78)	24.46(17.17, 31.75)	24.51(21.53, 27.49)	22.91(18.86, 26.97)	11.18(9.84, 12.52)	22.55(20.40, 24.70)	25.24(22.59, 27.88)
NPV (95% CI)	86.59(85.41, 87.77)	83.91(82.65, 85.18)	85.94(84.71, 87.17)	82.15(80.53, 83.76)	79.43(77.49, 81.37)	86.34(85.04, 87.65)	84.54(83.28, 85.79)	86.91(85.60, 88.22)	85.17(83.89, 86.46)	76.03(73.63, 78.44)	89.33 87.94, 90.73)	88.41(87.10, 89.72)
Sensitivity(95% CI)	18.56(11.85, 25.26)	1.37(0.00, 3.66)	18.21(13.58, 22.85)	26.80(24.27, 29.34)	35.05(32.76, 37.34)	30.93(27.42, 34.44)	6.19(2.10, 10.27)	37.11(33.77, 40.46)	17.87(14.17, 21.57)	45.02(42.90, 47.13)	61.86(59.35, 64.36)	49.48(46.44, 52.53)
Specificity(95% CI)	98.89(97.09, 100.00)	96.70(93.20, 100.00)	93.95(91.08, 96.81)	63.32(60.56, 66.08)	47.14(44.75, 49.54)	82.10(79.19, 85.01)	96.41(93.25, 99.56)	78.51(75.66, 81.35)	88.70(85.64, 91.76)	32.79(30.80, 34.79)	60.06(57.54, 62.58)	72.44(69.72, 75.16)

*Note*: Data reweighted to biopsied population. Note that CIN2 is treated as a false positive for the CIN3+ analysis.

Abbreviations: ASC‐H, atypical squamous cells—cannot rule out HSIL; ASC‐US, atypical squamous cells of undetermined significance; CI, confidence interval; CIN2, cervical intraepithelial neoplasia grade 2 [CIN2] or greater [CIN2+]; CIN3, cervical intraepithelial neoplasia grade 3 [CIN3] or greater [CIN3+]; hrHPV, high‐risk human papillomavirus; HSIL, high‐grade squamous intraepithelial lesion or greater [HSIL+]; LSIL, low‐grade squamous intraepithelial lesion; NPV, negative predictive value; PPV, positive predictive value.

^a^ Denominator excludes CIN3+ women (n = 459).

S5 scores for women not biopsied were similar to those biopsied with <CIN2 (median 0.61 vs 0.68 [unweighted], *P* = .70). Greater proportions of high S5 scores were also observed in women who were HPV16 positive compared to all other hrHPV types (*P* < .001, Figure S2). The S5 score was not significantly related to age (unadjusted *P* = .96, adjusted for histology *P* = .68), with a median value of 0.71 for women <30 years, 0.75 for women 30 to 45 years and 0.85 for those aged 46 or more years.

**FIGURE 2:**
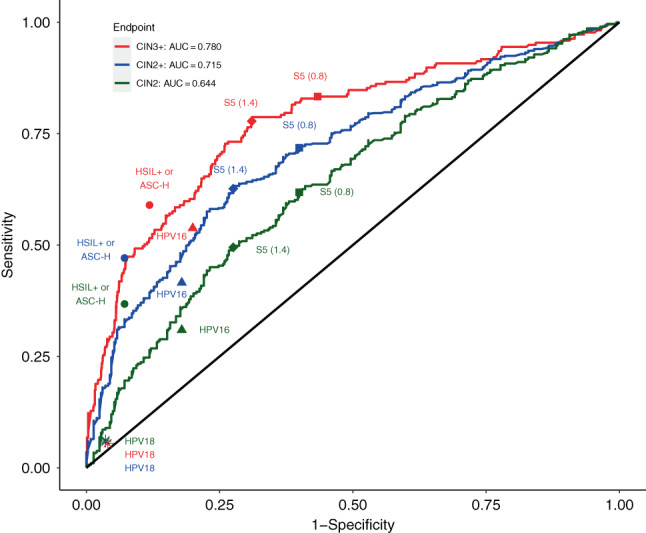
ROC curve for the S5 classifier for CIN3+ vs <CIN3, CIN2+ vs <CIN2 and CIN2 vs <CIN2 endpoints restricted to biopsied hrHPV positive women with abnormal cytology, re‐weighted to represent hrHPV positive biopsied women (n = 3797). See Table S1 for AUC values for other comparisons. Point estimates are also given for HSIL+ or ASC‐H cytology, HPV16 and HPV18 genotyping (separately) and S5 at 0.8 and 1.4 cut‐offs. Receiver operator characteristic (ROC) curves showing the diagnostic ability of S5 methylation for outcomes CIN3+, CIN2+ and CIN2 separately. Sensitivity and specificity are also plotted for HSIL+ or ASC‐H cytology, HPV16 and HPV18 genotyping (separately) and S5 at 0.8 and 1.4 cut‐offs. AUC is calculated for outcomes CIN3+, CIN2+ and CIN2. AUC, area under the curve; CIN2, cervical intraepithelial neoplasia grade 2 [CIN2] or greater [CIN2+]; CIN3, cervical intraepithelial neoplasia grade 3 [CIN3] or greater [CIN3+]; hrHPV, high‐risk human papillomavirus; HSIL, high‐grade squamous intraepithelial lesions or greater [HSIL+]

In Figure 2, S5 ROC curves are plotted for CIN3+, CIN2+ and CIN2, indicating clear differences between the groups in positivity rates for different cut‐off values. The AUC for CIN3+ was 0.780, indicating S5 provided good discrimination between CIN3+ and <CIN3. The AUC decreased to 0.715 for CIN2+ and to 0.644 for CIN2 alone vs <CIN2 (with CIN3+ cases omitted). AUC values between <CIN2, CIN2 and CIN3+ histopathology showed significant differences for all pairwise comparisons (*P* < .001, Table S1), with a greater difference between CIN3+ and <CIN2 (0.796) than for CIN3+ and <CIN3.

### 3.2 Comparison with other potential triage tests

The diagnostic accuracy of S5 methylation for women with abnormal cytology was compared to cytology and genotyping (with five groups; HPV16, HPV18, HPV16/18, HPV31/33 and other hrHPV) for CIN3+, CIN2+ and CIN2 endpoints (Table 2). Except for ASC‐US+ cytology, which by design occurred in almost all women in this population, sensitivity was substantially higher for S5 than for any other triage test. PPV and specificity increased with increasing cytological grade (Table 2). This is further illustrated in Figure 2, where the point estimates for sensitivity and specificity of HSIL+ and ASC‐H cytology, HPV16 and HPV18 genotyping, and S5 at the 0.8 and 1.4 cut‐offs are plotted, against the full S5 ROC curves.

HSIL+ and ASC‐H, taken together occurred in 17.54% of this ASC‐US+ referral population (666/3797, Table 2). Both showed high PPV values for CIN3+ (54.94% and 29.79%, respectively) and high specificity for <CIN2 (98.89% and 93.95%, respectively). When HSIL+ and ASC‐H were combined, the PPV for CIN3+ was 40.61%, and specificity for <CIN2 was 92.84%, which were higher than for S5. However their combined sensitivity for CIN3+ was substantially lower than seen for S5 (58.97% vs 83.33%, *P* < .001, Figure 2).

For women with abnormal cytology, 28.23% were HPV16/18 positive, 12.75% were HPV31/33 positive (and HPV16/18 negative) and 59.02% were positive only for other hrHPV types (Table 2). While HPV16/18 genotyping had a somewhat higher PPV than S5 (25.36% vs 20.87% for CIN3+, *P* = .005), its sensitivity for CIN3+ was 59.23%, which was similar to HSIL+ and ASC‐H combined (*P* = .89), but again much lower than for S5 (*P* < .001). Of note, HPV16/18 sensitivity was highly dominated by HPV16. For CIN3+, sensitivity was 53.78% for HPV16 but only 5.49% for HPV18 (Table 2). High concordance for HPV testing results was observed between Linear Array and the clinical HPV assay (Table S2).

### 3.3 Combining triage markers

Women with HSIL+ or ASC‐H cytology have a high PPV and very high specificity for CIN3+ thus not requiring further triage before colposcopy. For women with indeterminate or low‐grade cytology (ASC‐US or LSIL), both S5 and genotyping are informative. In this group (n = 3014) the PPV for CIN3+ was 5.69% overall; for S5 positivity it was 10.40% compared to 13.15% for HPV16/18 positivity and 8.22% for HPV31/33 positivity (Table 3).

**TABLE 3:** Positive predictive values (PPV) for combinations of HPV genotyping and S5 methylation restricted to biopsied women with abnormal cytology excluding high‐grade cytology (HSIL+, AGC and ASC‐H), for CIN3+, CIN2+ and CIN2 endpoints, re‐weighted to represent hrHPV positive biopsied women (n = 3014)

Marker		CIN3+ (n = 171)		CIN2+ (n = 498)		CIN2^a^ (n = 327)	
		n	PPV (95% CI)	n	PPV (95% CI)	n	PPV (95% CI)
All hrHPV positive	3014	171	5.69 (4.86, 6.51)	498	16.53 (15.20, 17.85)	327	11.49 (10.32, 12.67)
HPV16 positive	541	74	13.71 (10.81, 16.61)	161	29.82 (25.96, 33.67)	87	18.67 (15.13, 22.20)
HPV18 positive	120	13	10.61 (5.10, 16.12)	35	28.79 (20.68, 36.89)	22	20.34 (12.71, 27.96)
HPV16/18 positive	661	87	13.15 (10.57, 15.73)	196	29.63 (26.15, 33.11)	109	18.98 (15.77, 22.19)
HPV31/33 positive	359	30	8.22 (5.38, 11.06)	80	22.37 (18.06, 26.68)	51	15.41 (11.52, 19.31)
HPV16/18/31/33 positive	1020	116	11.42 (9.46, 13.37)	276	27.07 (24.35, 29.80)	160	17.68 (15.19, 20.16)
other hrHPV positive	1994	55	2.76 (2.04, 3.48)	222	11.13 (9.75, 12.51)	167	8.61 (7.36, 9.86)
S5 positive	1201	125	10.40 (8.68, 12.13)	310	25.82 (23.35, 28.30)	185	17.21 (14.96, 19.47)
S5 positive (≥1.4 cut‐off)	864	108	12.52 (10.31, 14.72)	252	29.12 (26.09, 32.15)	143	18.98 (16.18, 21.78)
S5 or HPV16 positive	1265	136	10.72 (9.01, 12.42)	335	26.51 (24.07, 28.94)	200	17.68 (15.46, 19.91)
S5 or HPV18 positive	1260	125	9.92 (8.26, 11.57)	323	25.62 (23.21, 28.03)	198	17.43 (15.22, 19.64)
S5 or HPV16/18 positive	1324	136	10.24 (8.60, 11.87)	348	26.28 (23.91, 28.65)	212	17.87 (15.69, 20.05)
S5 or HPV31/33 positive	1386	136	9.78 (8.21, 11.34)	341	24.58 (22.31, 26.84)	205	16.40 (14.35, 18.45)
S5 or HPV16/18/31/33 positive	1509	146	9.68 (8.19, 11.17)	379	25.08 (22.89, 27.26)	232	17.05 (15.05, 19.04)
S5 or other hrHPV positive	2706	150	5.55 (4.69, 6.42)	430	15.88 (14.51, 17.26)	280	10.94 (9.73, 12.15)

*Note*: Data re‐weighted to biopsied population.

Abbreviations: AGC, atypical glandular cells; ASC‐H, atypical squamous cells—cannot rule out HSIL; CI, confidence interval; CIN2, cervical intraepithelial neoplasia grade 2 [CIN2] or greater [CIN2+]; CIN3, cervical intraepithelial neoplasia grade 3 [CIN3] or greater [CIN3+]; hrHPV, high‐risk human papillomavirus; HSIL, high‐grade squamous intraepithelial lesion or greater [HSIL+]; PPV, positive predictive value.

^a^ Denominator excludes CIN3+ women (n = 171).

Excluding women with high‐grade cytology (HSIL+, ASC‐H or AGC), sensitivity of S5 for the remaining CIN3+ cases was 72.87% while for HPV16/18 positivity was 50.69% (Table S3). Considering combinations of either S5 or HPV genotype positivity, the sensitivity increased to 79.05% (*P* < .001) for S5 and/or HPV16/18 positivity, 79.05% (*P* < .001) for S5 and/or HPV31/33 positivity and 85.24% (*P* < .001) if S5 or any of these four genotypes were positive, with modest reductions in specificity. Combining S5 with all other hrHPV types increased sensitivity to 87.64% (*P* < .001), but specificity for <CIN2 was reduced to only 9.54%.

Importantly, hrHPV positive women with abnormal cytology, but excluding those with high‐grade cytology (HSIL+, AGC or ASC‐H; n = 3014), could avoid biopsy if negative for S5 where a CIN3+ PPV of 2.56% was observed (Table S4). However, of those S5 negative, only 6.78% were HPV16/18 positive, of which only 8.60% were CIN3+. For hrHPV positive women with abnormal cytology, excluding those with high‐grade cytology or HPV16/18 positivity, 28.18% of women were S5 positive, providing a PPV for CIN3+ of 7.33% and a sensitivity of 57.52% (Table S4).

## 4 DISCUSSION

Using data from the NMHPVPR, we investigated the clinical utility of the DNA methylation classifier S5 as a triage test for hrHPV positive women biopsied after routine cervical screening. The S5 methylation scores increased with increasing histopathology severity with a significant difference in median S5 scores for all increments in histology from <CIN2, CIN2 and CIN3/AIS to cancer. This is consistent with previous studies evaluating the S5 classifier, which found median S5 scores to be significantly increased for CIN3 and cancer cases.^15^, ^20^, ^29^, ^30^ The previously validated 0.8 cut‐off score provided higher detection rates for all histological outcomes compared to a 1.4 cut‐off. Generally, we seek to maximise the detection of cancers and CIN3, and minimise the detection of normal, CIN1 and CIN2; this process requires careful selection of the cut‐offs in a population‐dependent manner. Populations with higher HPV prevalence and less screened women require higher cut‐offs.^35^ While high positivity for women with negative and CIN1 diagnoses is undesirable, the sensitivity for CIN2 vs <CIN2 was greater for a 0.8 cut‐off, and there was a relatively small reduction in specificity compared to the cut‐off at 1.4. Importantly, our results support that S5 may be able to distinguish between CIN2 and CIN3/AIS/cancer, which has also been reported by our group previously.^16^ While many studies have shown very high sensitivity and specificity for cancer using S5, there were too few cancers cases in our sample (n = 8) to make any strong inferences.

In our study, for women with any abnormal cytology, the S5 classifier had substantially greater sensitivity for CIN3+ than HPV16/18 detection (83.33% vs 59.28%), and only a slightly lower PPV (20.87% vs 25.36%), consistent with the lower specificity, although the PPV for CIN3+ was well above the 5% level deemed to be appropriate for referral to colposcopy in the United States.^36^ Similar results were seen in the HPV FOCAL trial, where S5 methylation provided a CIN3 sensitivity of 93.2% and a specificity of 44.0% for <CIN2 compared to 86.4% and 52.0%, respectively, for combined abnormal cytology or HPV16/18 genotyping.^15^ Further, in women attending routine cervical screening in London, S5 showed greater sensitivity for CIN3+ than HPV16/18 genotyping (84% vs 58%), and similar specificity for <CIN2 (65% vs 71%).^20^

Other methylation markers have also been evaluated as triage tools in cervical screening and most have also shown increased sensitivity and specificity compared to HPV genotyping or cytology triage.^37^, ^38^, ^39^, ^40^ However, due to differences in gene panels used, and study designs, direct comparison of methylation classifiers is complicated. In a subset of samples from the POBASCAM trial, methylation of the promotor regions of *CADM1* and *MAL* achieved a sensitivity of 84.2% and specificity of 52.5%, which was very similar to that achieved by considering either abnormal cytology or HPV16/18 positivity as grounds for referral (sensitivity 84.2%, specificity 54.0%).^37^ In a worldwide study in women with invasive cervical cancer, the effectiveness of the QIAsure methylation assay (QIAGEN, Germany) for the *FAM19A4*/miR124‐2 genes showed very high sensitivity for cervical cancer,^26^, ^41^ with nearly all carcinomas, including rare histological types and hrHPV negative carcinomas identified.^42^ This provides promise that women negative for methylation markers have a very low chance of having cervical cancer. Another DNA methylation biomarker panel, the GynTect test (Oncgnostics, Germany)^43^ has shown modest CIN3+ sensitivity (64.8%) and good specificity (94.6%).^44^

In our study, S5 provided good discrimination between CIN3+ and <CIN3 biopsies, consistent with findings from previous studies.^15^, ^20^ In a UK based screening study of hrHPV positive women, the AUC for CIN3+ was 0.84^20^ and in the HPV FOCAL trial it was 0.83.^15^ We observed even greater discrimination between CIN3+ and low‐grade (CIN1) or negative biopsies.

The biological and clinical meaning of a CIN2 diagnosis remains not well understood, and the value of detecting CIN2 is uncertain.^45^ Most CIN2 cases regress naturally making it difficult to decide on the best course of action, especially in younger women.^4^ CIN2 diagnoses also suffer from a lack of reproducibility.^46^ We found that there was a significant difference in AUCs for CIN2 and CIN3+ endpoints, supporting that CIN2+ as a clinical endpoint is a composite of different clinical entities and is potentially misleading. The performance of S5 to detect CIN2 was low compared to CIN2+ indicating the latter is strongly influenced by CIN3+. Using S5 methylation as a triage test may have an advantage over genotyping or cytology in that it may have the ability to better differentiate between progressive vs regressive CIN2, where optimal management may be different.^16^ This is an important area for future research.

There is clear evidence, seen here and elsewhere, that women with HSIL+ and ASC‐H cytology should be referred to colposcopy. However, for women with lesser cytologic abnormalities (ASC‐US and LSIL), appropriate management is less clear. In this population, the PPV for CIN3+ was high enough for women positive for either HPV16/18 (13.15%), HPV31/33 (8.22%) or S5 methylation (10.40%) that immediate referral to colposcopy would be recommended based on the previously suggested 5% threshold.^36^ However, while the PPV for HPV16 or HPV18 positivity was similar to that for S5 positivity, sensitivity was much greater for S5 (72.87% vs 50.69%) among those with lesser cytologic abnormalities. This greater sensitivity is important given that some HPV tests now routinely provide partial genotyping information. We found substantial improvements in sensitivity when performing S5 methylation in women who were HPV16/18 negative. Data from the FRIDA study has also shown the benefit of using S5 as a highly sensitive second triage in women with ASC‐US+ cytology who were HPV16/18 negative, where a reduction of 43% in colposcopy referrals would have been observed if S5 was used.^29^ While it can be argued that additional triage tests increase the cost of screening, it need only be done selectively in those where management is uncertain using conventional triage tests, and it is likely that the reduction in colposcopy referral would offset this cost.

As noted earlier, methylation is a quantitative measure and has the potential advantage over other tests to be more precise in guiding a flexible management decision about immediate colposcopy referral vs short term repeat testing. Also, methylation can be automated, and performed on multiple specimen types, including vaginal self‐samples and urine; allowing for reflex testing as compared to cytology which requires women to return to the clinic to provide additional specimens. However, while the use of methylation as a biomarker of cervical pre‐cancer remains promising, the challenges of pyrosequencing and the slowness of commercial development has failed to drive methylation to the forefront of clinical management. Further, as the coverage of HPV vaccination grows, more vaccinated women will be screening and the need for surveillance in these women will decrease. Thus, cost‐effectiveness analysis will require re‐evaluation over time.

While the NMHPVPR provided a large population‐based cohort of women biopsied after a routine screening test, there are some limitations of our study. Our study was an evaluation in women who received cervical screening and follow‐up in a routine clinical practice setting, thus, were subject to more varied clinical management and follow‐up vs what would occur in standardised clinical trials. In addition, almost all biopsied women had abnormal cytology, and a substantial number of HPV16/18 positive, cytology negative women would not have been identified or biopsied during the period of our study, as co‐testing was only used in about 50% of screening tests at that time.^47^ Therefore, conclusions can only be reliably drawn for hrHPV positive women with abnormal cytology. The performance of cytology and to a lesser extent also HPV16/18 genotyping is subject to selection bias, as it is well known that referral bias can inflate the performance characteristics of the tests that are the basis of management decisions.^48^ Further studies are needed for hrHPV positive women with negative cytology and to follow‐up women with abnormal cytology or hrHPV who did not attend colposcopy within 12 months. Including more cases of cancer would also have been of value. Linear Array was the genotyping assay used in the NMHPVPR for research purposes. Although Linear Array is not a Food and Drug Administration (FDA) approved assay, it has shown good correlation with gold‐standard methods,^49^, ^50^ and strong concordance with clinical HPV data in our study when available (Table S2). Nevertheless, confirmation of these results in another screening population where the HPV test was performed by an assay approved by the FDA is desirable.

## 5 CONCLUSIONS

While HPV vaccination is likely to help reduce the incidence of HPV related disease, the full benefits of this are still decades away. Even in favourable circumstances, vaccination coverage is rarely above 70%, so cervical screening will still be needed. However, disease rates and HPV16/18 infections will be lower in vaccinated populations, so highly specific triage tests become even more important. In this regard it is encouraging to note that S5 testing of HPV positive women who were not positive for HPV16/18 had a relatively good PPV and sensitivity for CIN3+. Methylation of many different genes and panels has shown promise as triage tests for cervical pre‐cancer, especially in hrHPV positive women. S5 methylation provided a similar PPV and significantly greater sensitivity for CIN3+ than HPV genotyping or cytology triage, safely enabling a reduction in the number of unnecessary colposcopy referrals. The S5 classifier measured from a LBC specimen has also shown an ability to better distinguish between <CIN2, CIN2 and CIN3+ in subsequent biopsies, a finding of importance for managing CIN2, given the complexity and uncertainty associated with this diagnosis.

## AUTHOR CONTRIBUTIONS

The work reported in the article has been performed by the authors, unless clearly specified in the text. Attila T Lorincz, Jack Cuzick, Belinda Nedjai and Cosette M Wheeler were involved in the conceptualization. Rachael Adcock, Dorota Scibior‐Bentkowska, Rawinder Banwait, Norah Torrez‐Martinez and Michael Robertson were involved with the Data curation. Rachael Adcock, Belinda Nedjai and Jack Cuzick were involved in the Formal Analysis. Belinda Nedjai, Attila T Lorincz, Jack Cuzick and Cosette M Wheeler were involved in the Funding acquisition. All authors (Rachael Adcock, Belinda Nedjai, Attila T Lorincz, Dorota Scibior‐Bentkowska, Rawinder Banwait, Norah Torrez‐Martinez, Michael Robertson, Jack Cuzick and Cosette M Wheeler) were involved in the Investigation. Rachael Adcock, Belinda Nedjai, Attila T Lorincz, Jack Cuzick and Cosette M Wheeler were involved in the Methodology. Belinda Nedjai, Attila T Lorincz and Cosette M Wheeler were involved in the Resources and Project administration. Belinda Nedjai, Attila T Lorincz, Jack Cuzick and Cosette M Wheeler were involved in the supervision. Dorota Scibior‐Bentkowska, Rawinder Banwait and Norah Torrez‐Martinez were involved in the validation. Belinda Nedjai, Rachael Adcock, Attila T Lorincz, Jack Cuzick and Cosette M Wheeler were involved in Writing ‐ original draft. All authors were involved in the Writing ‐ review & editing.

## CONFLICT OF INTEREST

Jack Cuzick and Cosette M. Wheeler have received funds from grants, cooperative agreements or subcontracts related to cervical screening and triage through their respective institutions. Jack Cuzick reports grants and personal fees from Hologic, and grants from Qiagen, Becton Dickinson (BD) and Gene First, all outside the submitted work. Cosette M Wheeler reports receiving reagents and equipment from Roche Molecular Systems, Roche/Ventana Medical Systems, and Hologic and research support from BD and Hologic all through her institution and outside of the submitted work, and personal fees from BD also outside of the submitted work. Rachael Adcock, Belinda Nedjai, Attila T Lorincz, Dorota Scibior‐Bentkowska, Rawinder Banwait, Norah Torrez‐Martinez and Michael Robertson have no conflicts.

## ETHICS STATEMENT

Our study was approved by the University of New Mexico Human Research Review Committee (HRRC). Informed consent was waived by the HRRC.

## Supporting information


**Appendix S1** Supporting Information.

## Data Availability

Data supporting the investigation reported here can be made available in de‐identified form subject to establishing a data use agreement with the University of New Mexico Health Sciences Center (UNM‐HSC). For further information contact the UNM‐HSC Sponsored Projects Office https://hsc.unm.edu/financialservices/preaward/. Further information is also available from the corresponding author upon request.
