# Supplementary material for: DNA methylation testing with S5 for triage of high‐risk HPV positive women
Source: Int J Cancer. 2022 May 24;151(7):993–1004. doi: 10.1002/ijc.34050 (PMC9543033; doi:10.1002/ijc.34050)
Supplement: Supplementary file 1 — Appendix S1 Supporting Information. [file IJC-151-993-s001.pdf]

DNA methylation testing with S5 for triage of high-risk HPV positive women

Rachael Adcock, Belinda Nedjai, Attila Lorincz, Dorota Scibior-Bentkowska, Rawinder Banwait, Norah Torrez-Martinez, Michael Robertson, Jack Cuzick, Cosette M Wheeler on behalf of the New Mexico HPV Pap Registry Steering Committee

<u>Table of Contents</u>

Supplementary Table S1; Area under the curve (AUC) and 95% Confidence Intervals for comparisons restricted to <CIN2, CIN2 and CIN3+ endpoints for hrHPV positive women with abnormal cytology, re-weighted to represent hrHPV positive biopsied women (n=3797). All AUC values had P<0.001 vs no difference (AUC = 0.500).

Supplementary Table S2: Concordance between clinical HPV DNA data and research use of Linear Array HPV genotyping for the S5 sample of women with screening cytology and cervical biopsy within 12 months (n=798).

Supplementary Table S3: Sensitivity and specificity for combinations of HPV genotyping and S5 methylation restricted to biopsied women with abnormal cytology excluding high-grade cytology (HSIL+, AGC and ASC-H), for CIN3+, CIN2+ and CIN2 endpoints, re-weighted to represent hrHPV positive biopsied women (n=3014).

Supplementary Table S4: Positive Predictive Value (PPV) and sensitivity of a second triage test restricted to hrHPV positive women with abnormal cytology excluding high-grade cytology (HSIL+, AGC and ASC-H), for CIN3+, CIN2+ and CIN2 endpoints, re-weighted to represent hrHPV positive biopsied women (n=3014).

Supplementary Figure S1: Consort diagram

Supplementary Figure S2: Histogram of S5 score by genotype (HPV16, HPV18, HPV31/33 and other hrHPV) for hrHPV positive women with screening cytology. Percentages add to 100% in each HPV group.

Supplementary Table S1: Area under the curve (AUC) and 95% Confidence Intervals for comparisons restricted to <CIN2, CIN2 and CIN3+ endpoints for hrHPV positive women with abnormal cytology, re-weighted to represent hrHPV positive biopsied women (n=3797). All AUC values had P<0.001 vs no difference (AUC = 0.500).

Comparison	AUC (95% CI)
<CIN2 vs CIN2	0.644 (0.596, 0.691)
<CIN2 vs CIN2+	0.715 (0.676, 0.753)
<CIN2 vs CIN3	0.790 (0.746, 0.843)
<CIN2 vs CIN3+	0.796 (0.754, 0.839)
CIN2 vs CIN3	0.684 (0.635, 0.733)
CIN2 vs CIN3+	0.693 (0.646, 0.741)
<CIN3 vs CIN3	0.773 (0.730, 0.816)
<CIN3 vs CIN3+	0.780 (0.739, 0.821)

hrHPV: high-risk Human Papillomavirus; AUC: Area Under the Curve; CI: Confidence Interval; CIN2: Cervical Intraepithelial Neoplasia grade 2 [CIN2] or greater [CIN2+]; CIN3: Cervical Intraepithelial Neoplasia grade 3 [CIN3] or greater [CIN3+]

Supplementary Table S2: Concordance between clinical HPV DNA data and research use of Linear Array HPV genotyping for the S5 sample of women with screening cytology and cervical biopsy within 12 months (n=798).

		**Clinical HPV**						
		HPV16	HPV18	HPV16/18 negative, other hrHPV positive	hrHPV positive, no genotyping	hrHPV negative	No clinical hrHPV data	Total
**Linear Array**	HPV16	**185**	1	1	11	0	66	264
	HPV18	0	**38**	2	2	0	5	47
	Other hrHPV	2	0	**352**	24	7	102	487
	Total	187	39	355	37	7	173	798

Numbers in **bold** are concordant.

hrHPV: high-risk Human Papillomavirus

Clinical HPV testing was performed using the Cobas HPV assay (93%), the Aptima HPV assay (2%) and the Hybrid Capture 2 assay (6%)

Supplementary Table S3: Sensitivity and specificity for combinations of HPV genotyping and S5 methylation restricted to biopsied women with abnormal cytology excluding high-grade cytology (HSIL+, AGC and ASC-H), for CIN3+, CIN2+ and CIN2 endpoints, re-weighted to represent hrHPV positive biopsied women (n=3014).

Marker (n positive)		CIN3+ (n=171)			CIN2+ (n=498)			CIN2[a] (n=327)		
		n	Sensitivity (95% CI)	Specificity (95% CI)	n	Sensitivity (95% CI)	Specificity (95% CI)	n	Sensitivity (95% CI)	Specificity (95% CI)
HPV16 positive	541	74	43.28 (39.10, 47.45)	83.58 (40.46, 86.70)	161	32.38 (28.44, 36.33)	84.91 (81.89, 87.93)	87	26.67 (22.66, 30.68)	84.91 (81.66, 88.16)
HPV18 positive	120	13	7.42 (2.73, 12.11)	96.23 (92.82, 99.64)	35	6.92 (2.38, 11.47)	96.61 (93.37, 99.85)	22	6.67 (1.94, 11.39)	96.61 (93.18, 100.04)
HPV16/18 positive	661	87	50.69 (46.88, 54.50)	79.81 (76.75, 82.87)	196	39.31 (35.58, 43.03)	81.52 (78.56, 84.48)	109	33.33 (29.48, 37.19)	81.52 (78.34, 84.69)
HPV31/33 positive	359	30	17.24 (13.33, 21.14)	88.40 (85.09, 91.71)	80	16.13 (12.33, 19.94)	88.91 (85.67, 92.16)	51	15.56 (11.64, 19.47)	88.91 (85.53, 92.30)
HPV16/18/31/33 positive	1020	116	67.93 (65.07, 70.79)	68.21 (65.35, 71.07)	276	55.44 (52.39, 58.49)	70.43 (67.63, 73.23)	160	48.89 (45.63, 52.15)	70.43 (67.46, 73.41)
other hrHPV positive	1994	55	32.07 (30.02, 34.12)	31.79 (29.74, 33.83)	222	44.56 (42.38, 46.74)	29.57 (27.56, 31.57)	167	51.11 (48.89, 53.34)	29.57 (27.54, 31.60)
S5 positive	1201	125	72.87 (70.36, 75.39)	62.16 (59.41, 64.90)	310	62.24 (59.50, 64.99)	64.60 (61.90, 67.31)	185	56.67 (53.71, 59.63)	64.60 (61.74, 67.46)
S5 positive (≥1.4 cut-off)	864	108	63.05 (59.84, 66.27)	73.42 (70.48, 76.37)	252	50.48 (47.15, 53.82)	75.67 (72.81, 78.53)	143	43.89 (40.35, 47.43)	75.67 (72.61, 78.73)
S5 or HPV16 positive	1265	136	79.05 (76.81, 81.30)	60.28 (57.59, 62.98)	335	67.29 (64.70, 69.87)	63.06 (60.40, 65.72)	200	61.11 (58.27, 63.95)	63.06 (60.25, 65.88)
S5 or HPV18 positive	1260	125	72.87 (70.42, 75.33)	60.07 (57.37, 62.78)	323	64.79 (62.16, 67.43)	62.75 (60.08, 65.42)	198	60.56 (57.71, 63.40)	62.75 (59.94, 65.56)
S5 or HPV16/18 positive	1324	136	79.05 (76.86, 81.25)	58.20 (55.54, 60.85)	348	69.84 (67.36, 72.31)	61.21 (58.59, 63.83)	212	65.00 (62.29, 67.71)	61.21 (58.44, 63.98)
S5 or HPV31/33 positive	1386	136	79.05 (76.91, 81.20)	56.01 (53.40, 58.62)	341	68.38 (65.93, 70.83)	58.45 (55.85, 61.04)	205	62.78 (60.10, 65.46)	58.45 (55.72, 61.18)

S5 or HPV16/18/31/33 positive	1509	146	85.24 (83.45, 87.03)	52.05 (49.53, 54.57)	379	75.97 (73.82, 78.13)	55.06 (52.55, 57.57)	232	71.11 (68.71, 73.52)	55.06 (52.42, 57.70)
S5 or other hrHPV positive	2706	150	87.64 (86.40, 88.88)	10.11 (8.97, 11.24)	430	86.27 (84.98, 87.57)	9.54 (8.44, 10.65)	280	85.56 (84.19, 86.92)	9.54 (8.41, 10.68)

Data re-weighted to biopsied population. Note that CIN2 is treated as a false positive for the CIN3+ analysis.

[a] Denominator excludes CIN3+ women (n=171).

hrHPV: high-risk Human Papillomavirus; CIN2: Cervical Intraepithelial Neoplasia grade 2 [CIN2] or greater [CIN2+]; CIN3: Cervical Intraepithelial Neoplasia grade 3 [CIN3] or greater [CIN3+]; PPV: Positive Predictive value; CI: Confidence Interval; HSIL: High-grade Squamous Intraepithelial Lesion or greater [HSIL+]; AGC: Atypical Glandular Cells; ASC-H: Atypical Squamous Cells – cannot rule out HSIL.

Supplementary Table S4: Positive Predictive Value (PPV) and sensitivity of a second triage test restricted to hrHPV positive women with abnormal cytology excluding high-grade cytology (HSIL+, AGC and ASC-H), for CIN3+, CIN2+ and CIN2 endpoints, re-weighted to represent hrHPV positive biopsied women (n=3014).

**1st triage test**	**2nd triage test**	**n**	**CIN3+**			**CIN2+**			**CIN2**[a]		
			**n**	**PPV (95% CI)**	**Sensitivity (95% CI)**	**n**	**PPV (95% CI)**	**Sensitivity (95% CI)**	**n**	**PPV (95% CI)**	**Sensitivity (95% CI)**
HPV16 negative (n=2473)	-		97	3.93 (3.17, 4.70)	56.72 (54.77, 58.68)	337	13.62 (12.27, 14.97)	67.62 (65.77, 69.46)	240	10.08 (8.87, 11.30)	73.33 (71.56, 75.11)
	S5 positive	724	61	8.48 (6.45, 10.51)	63.08 (59.56, 66.59)	174	24.03 (20.92, 27.14)	51.62 (47.98, 55.26)	113	16.99 (14.13, 19.85)	46.97 (43.17, 50.77)
	S5 positive (1.4 cut-off)	406	44	10.97 (7.93, 14.01)	45.76 (40.92, 50.61)	119	29.32 (24.89, 33.75)	35.31 (30.65, 39.96)	74	20.61 (16.44, 24.78)	31.06 (26.29, 35.83)
HPV16/18 negative (n=2353)	-		85	3.59 (2.84, 4.34)	49.31 (47.29, 51.33)	302	12.85 (11.50, 14.20)	60.69 (58.72, 62.67)	218	9.60 (8.39, 10.81)	66.67 (64.73, 68.61)
	S5 positive	663	49	7.33 (5.35, 9.32)	57.52 (53.76, 61.28)	152	22.94 (19.74, 26.14)	50.30 (46.50, 54.11)	103	16.84 (13.88, 19.80)	47.50 (43.55, 51.45)
	S5 positive (1.4 cut-off)	369	38	10.34 (7.24, 13.45)	45.13 (40.05, 50.21)	105	28.55 (23.94, 33.16)	34.83 (29.97, 39.69)	67	20.31 (15.98, 24.65)	30.83 (25.86, 35.81)
S5 negative (n=1813)	-		47	2.56 (1.84, 3.29)	27.13 (25.08, 29.17)	188	10.37 (8.97, 11.77)	37.76 (35.53, 39.99)	142	8.01 (6.75, 9.28)	43.33 (41.02, 45.64)
	HPV16 positive	64	11	16.58 (7.46, 25.70)	22.79 (12.50, 33.07)	25	39.31 (27.34, 51.29)	13.35 (5.01, 21.69)	15	27.25 (15.29, 39.20)	10.26 (2.11, 18.40)
	HPV16/18 positive	123	11	8.60 (3.65, 13.56)	22.79 (15.38, 30.19)	38	30.71 (22.56, 38.86)	20.11 (13.03, 27.19)	27	24.19 (16.28, 32.10)	19.23 (11.95, 26.51)

Data re-weighted to biopsied population.

[a] Denominator excludes CIN3+ women.

hrHPV: high-risk Human Papillomavirus; CIN2: Cervical Intraepithelial Neoplasia grade 2 [CIN2] or greater [CIN2+]; CIN3: Cervical Intraepithelial Neoplasia grade 3 [CIN3] or greater [CIN3+]; PPV: Positive Predictive value; CI: Confidence Interval; HSIL: High-grade Squamous Intraepithelial Lesion or greater [HSIL+]; AGC: Atypical Glandular Cells; ASC-H: Atypical Squamous Cells – cannot rule out HSIL.

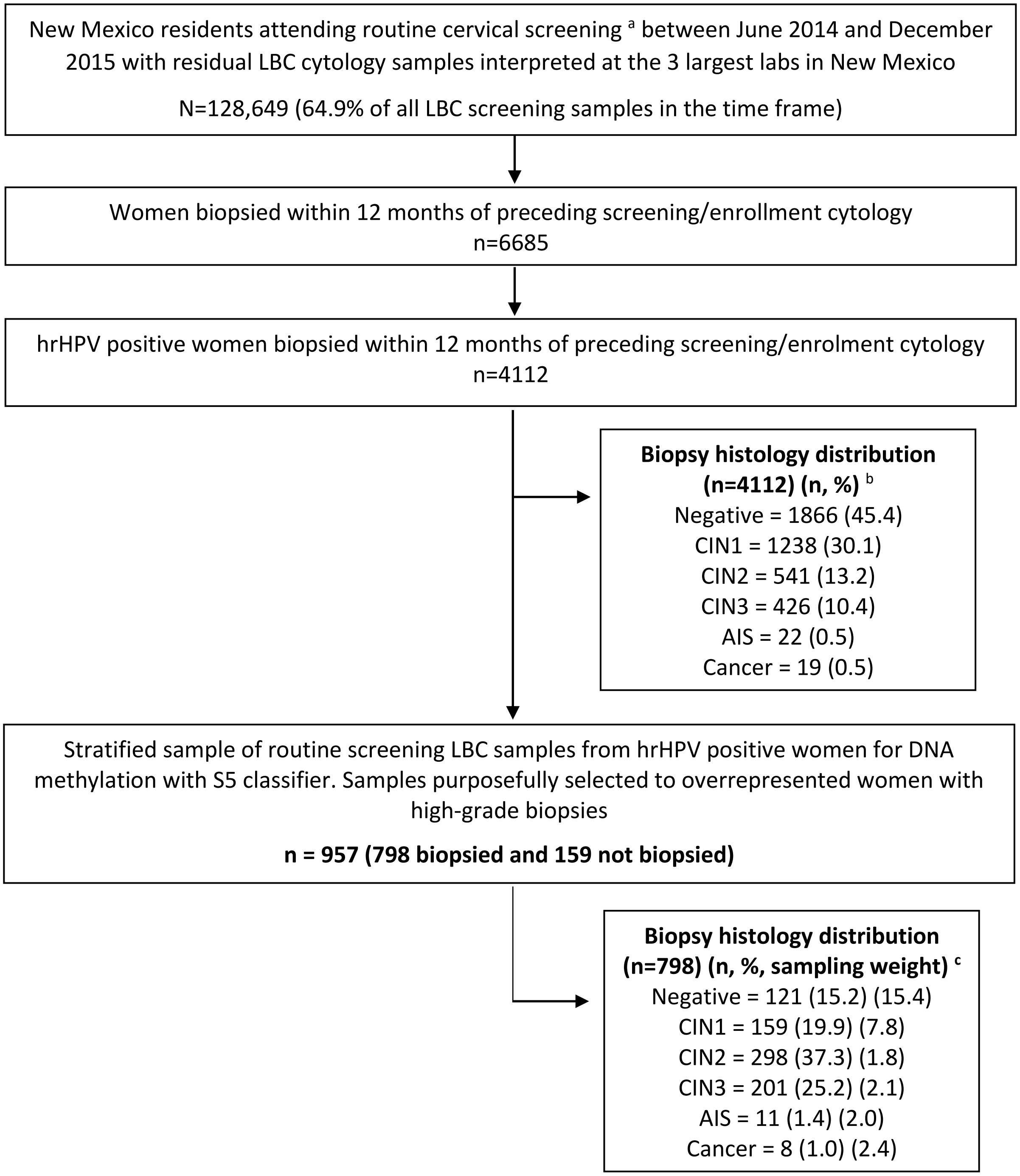

Supplementary Figure S1: Consort diagram

[a] Screening cytology defined as cytology with no prior hysterectomy and no prior cytology, HPV or biopsy in the past 300 days; [b] Highest biopsy diagnoses within 12 months of screening cytology restricted to earliest cytology chronologically per woman; [c] Samples selected to over represent high-grade disease.

hrHPV: high-risk Human Papillomavirus; LBC: Liquid Based Cytology; CIN1: Cervical Intraepithelial Neoplasia grade 1; CIN2: Cervical Intraepithelial Neoplasia grade 2; CIN3 Cervical Intraepithelial Neoplasia grade 3; AIS: Adenocarcinoma *in situ*.

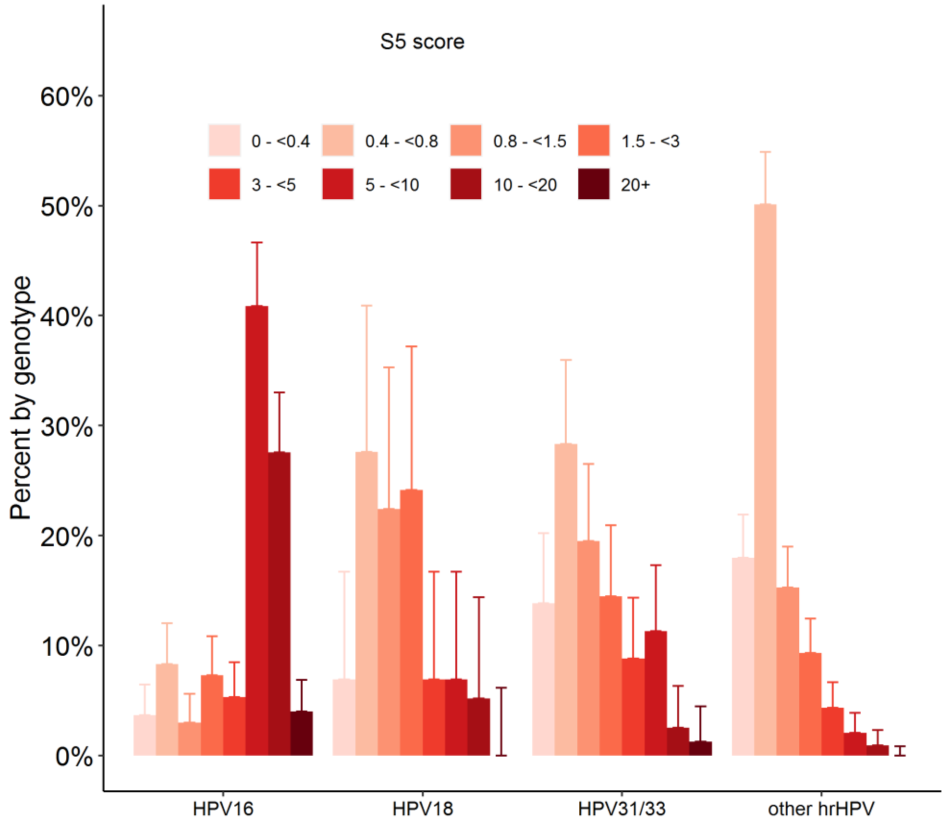

Supplementary Figure S2: Histogram of S5 score by genotype (HPV16, HPV18, HPV31/33 and other hrHPV) for hrHPV positive women with screening cytology. Percentages add to 100% in each HPV group.

hrHPV: high-risk Human Papillomavirus

A histogram showing the distribution of S5 scores (categorised) by HPV genotype (HPV16, HPV18, HPV31/33, other hrHPV types). Upper 95% confidence intervals are plotted. Percentage of S5 score sum to 100% within each genotype group. Higher risk HPV types are associated with greater proportions of higher S5 scores.
